# Joint Effect of Childhood Abuse and Family History of Major Depressive Disorder on Rates of PTSD in People with Personality Disorders

**DOI:** 10.1155/2012/350461

**Published:** 2012-04-03

**Authors:** Janine D. Flory, Rachel Yehuda, Vincent Passarelli, Larry J. Siever

**Affiliations:** ^1^Department of Psychiatry, Mount Sinai School of Medicine (MSSM), One Gustave L. Levy Place, NY 10029, USA; ^2^Department of Psychiatry, James J. Peters Veterans Affairs Medical Center, Bronx, NY 10468, USA

## Abstract

*Objective*. Childhood maltreatment and familial psychopathology both lead to an increased risk of the development of posttraumatic stress disorder (PTSD) in adulthood. While family history of psychopathology has traditionally been viewed as a proxy for genetic predisposition, such pathology can also contribute to a stress-laden environment for the child. *Method*. Analyses were conducted to evaluate the joint effect of childhood abuse and a family history of major depressive disorder (MDD) on diagnoses of PTSD and MDD in a sample of 225 adults with DSM-IV Axis II disorders. *Results.* Results showed that the rate of PTSD in the presence of both childhood abuse and MDD family history was almost six-fold (OR = 5.89, *P* = .001) higher relative to the absence of both factors. In contrast, the rate of MDD in the presence of both factors was associated with a nearly three-fold risk relative to the reference group (OR = 2.75, *P* = .01). *Conclusions*. The results from this observational study contribute to a growing understanding of predisposing factors for the development of PTSD and suggest that joint effects of family history of MDD and childhood abuse on PTSD are greater than either factor alone.

## 1. Introduction

Trauma exposure is a necessary condition for the development of posttraumatic stress disorder (PTSD) but there is increasing recognition that trauma exposure alone does not necessarily result in sufficient symptomatology to result in diagnosis. In fact, the observation from epidemiological samples that trauma exposure is highly prevalent but that PTSD is relatively rare [1–3] has generated research aimed at identifying environmental and genetic risk factors that can explain why only some people who are exposed to trauma will develop PTSD. Childhood maltreatment is unquestionably a potent environmental antecedent for the development of adult onset PTSD (e.g., [4, 5]), either resulting from the maltreatment itself or by increasing risk for exposure to subsequent traumatic events [6, 7].

With respect to genetic risk, it has not been obvious that molecular genetic factors are implicated in association with PTSD until recently, but there is increasing support for such a view. The genetic contribution to PTSD has been estimated at 30–40%, based on differences in concordance rates of the diagnosis between monozygotic and dizygotic twins [8–10]. And while familial psychopathology has traditionally been viewed as a proxy variable for genetic risk [11], few family studies of PTSD have been conducted. This is in part, due to the fact that the diagnosis is a relatively recent one, only appearing in the Diagnostic and Statistical Manual in 1980. Additionally, because any underlying genetic vulnerability for PTSD requires exposure to trauma for diagnosis, choosing a comparison group is not straightforward. Certain types of exposure may themselves be associated with genetic risk [9], and comparison subjects may possess the genetic vulnerability for PTSD but lack exposure to a traumatic event. See Yehuda et al. [12] for a discussion of the difficulties of conducing genetic studies of PTSD. Despite these limitation, however, some evidence suggests that a family history of mood disorders increases risk for PTSD [13, 14].

Although there has been an active interest in the interaction of molecular genetic and environmental factors (e.g., and gene-by-environment interaction studies) [15, 16], no studies have examined the joint impact of family history of psychopathology and childhood maltreatment. Accordingly, the current analyses were conducted to examine the joint (or moderating) effect of familial depression and childhood abuse on the rate of PTSD in a sample of people with DSM-IV Axis II disorders, a sample that is “enriched” for risk factors of PTSD. That is, people with DSM-IV Axis II disorders frequently carry comorbid PTSD diagnoses [17, 18], and childhood maltreatment is prospectively associated with the development of various personality disorders [19]. For comparison purposes, we also examined the rates of MDD in probands in association with these two risk factors.

## 2. Methods

### 2.1. Participants

Data for the current analyses were derived from 225 adult probands between the ages of 18 and 65 who participated in the Mount Sinai Mood and Personality Disorder (PD) research program between October 2004 and May 2009. The aim of the research program is to study neurobiological correlates of DSM-IV Axis II disorders, and research participants are recruited principally through newspaper and related media advertisements and postings on message boards and the internet. Inclusion criteria for the research program include absence of lifetime history of schizophrenia or bipolar disorder, current and significant medical illness, or a current diagnosis of major depression, substance abuse or dependence. Individuals with a history of past drug dependence are excluded from participation (e.g., IV or crack cocaine dependence). Following participation in an umbrella protocol that included completion of diagnostic interviews for the assessment of DSM-IV Axis I and Axis II psychopathology and self-report questionnaires, individuals were approached to participate in a second research protocol that included a family history interview. Participants provided written informed consent, and the protocols were approved by the Mount Sinai School of Medicine and James J. Peters VAMC Institutional Review Boards.

Participants ranged in age from 18 to 65 (X = 35.89, sd = 11.76), and approximately half of the sample was male (52%). With regard to racial and ethnic distribution, 55% of the sample was white, 28% was black, 10% was Asian, and 8% reported bi- or multi-racial ancestry. Twenty-four percent identified themselves as Hispanic. The sample was relatively well educated, with over half possessing a bachelor's degree or higher. The majority of the participants were not married or living with a partner (67%). The distribution of rater assigned DSM-IV Axis II diagnoses included (in order of highest to lowest): borderline PD = 110 (49%), obsessive compulsive PD = 90 (40%), paranoid PD = 82 (36%), schizotypal PD = 72 (32%), avoidant PD = 66 (29%), narcissistic PD = 44 (20%), antisocial PD = 24 (11%), dependent PD = 15 (7%), histrionic PD = 10 (4%), and schizoid PD = 5 (2%). These percentages sum to more than 100 because of comorbidity across diagnoses. The number of diagnoses met ranged from 1 to 6.

### 2.2. Measures

#### 2.2.1. DSM-IV Axis I and II Disorders

 Participants were interviewed for the presence of DSM-IV Axis I disorders using a semistructured interview which included the Structured Clinical Interview for DSM-IV (SCID-I; [20]). This measure was modified to include a full assessment of trauma history using the Trauma History Questionnaire [21]. This interview is based on the high-magnitude stressor questionnaire used in the DSM-IV field trials and is designed to cover a broad range of events that could be considered potentially traumatic, including those related to crime, general trauma, and physical and sexual assault. Participants were queried about 23 events and if they had experienced an event, they provided additional information including the number of times it occurred, the age when it occurred, and the emotional impact of the event, rated on a scale of 1 to 5. This measure has been shown to have good test-retest stability [21] and has been used with a wide range of individuals [22, 23]. Responses to the interview were used to identify potential Criterion A events for the assessment of a life-time history of PTSD. The presence of DSM Axis II disorders was assessed using the Structured Interview for DSM-IV PD (SIDP-IV, [24]). The majority of the diagnostic interviews (approximately 95%) were conducted by one of three doctoral level clinical psychologists and adjudicated for consensus with a licensed clinical psychologist.

#### 2.2.2. Family History Interview

 A modified Family Interview for Genetic Studies (FIGSs) [25] was used to assess the presence of MDD, alcohol and drug use disorders and characteristics of Borderline PD in adult first-degree relatives of the 225 probands. Administration of the FIGS began with the drawing of family pedigree that included only first-degree relatives over the age of 18. The proband was then asked a series of general screening questions and based on these responses, the interviewer completed symptom checklists for each of the four disorders named above. To protect confidentiality of the family members, probands were asked to name the total number of 1st-degree relatives in their family and then the total number who met that feature of the disorder without identifying a specific family member (e.g., “out of the four members of your family, how many had problems with depression?”). Family history interviews were conducted by trained interviewers who were blind to Axis I and II diagnostic status of the probands. The symptom checklists derived from the interviews were based on DSM-IV criteria and were coded and reviewed for consensus by a licensed clinical psychologist, who was also blind to the proband's diagnostic profile. To create family history variables for analysis, a dichotomous variable was created to distinguish probands who reported a positive family history of depression (i.e., any first-degree relative with MDD) from those reporting no family history of MDD. For MDD, a dichotomized family history positive score is comparable to family history density scores that account for family size [11]. Forty-four percent of the sample reported a positive family history of MDD (*n* = 98). Depression family history could not be determined from three probands.

#### 2.2.3. Childhood Physical Abuse

 The Childhood Trauma Questionnaire (CTQ) [26, 27] is comprised of 25 questions that ask individuals to record their impressions of childhood physical abuse, physical neglect, sexual abuse, emotional abuse, and emotional neglect, using a 5-point Likert-type scale (1: never true, 2: rarely true, 3: sometimes true, 4: often true, and 5: very often true). For the current analyses, the distribution of the total CTQ score was divided into tertiles, and values that fell in the upper tertile were coded 1 to reflect “high likelihood of childhood abuse” and those in the lower and middles tertiles were coded 0 to reflect “low likelihood of childhood abuse.” A 3-item Minimization/Denial subscale is also administered for detecting socially desirable responses that might reflect false negative trauma reports, for example, “I had the perfect childhood.” For these items, 1 point is scored for each item endorsed with a score of 5 (very often true), while all other responses are scored 0. The total score ranges from 0 to 3.

### 2.3. Statistical Analyses

 Logistic regression analyses were used to examine the joint effect of family history of MDD and childhood abuse (i.e., multiplicative interaction) on proband diagnoses of PTSD, and for comparison purposes, diagnoses of MDD. For these analyses, dummy codes were used to create four groups, including people who reported (1) no family history of MDD and low likelihood of childhood abuse (reference group; *n* = 90); (2) only high likelihood of childhood abuse (*n* = 34); (3) only family history of MDD (*n* = 56); (4) family history of MDD and high likelihood of childhood abuse (*n* = 42). Because the proband's gender is a potential confounder, this variable was entered on the first step of the logistic regression equation.

## 3. Results

Table 1 presents the CTQ scale scores for people who were positive versus negative for a family history of MDD. Consistent with the view that family history of MDD can reflect an environment laden with stress exposure; results showed that people with a positive family history reported higher levels of emotional abuse, sexual abuse, and physical neglect. People without a family history of MDD were not more likely to report socially desirable responses than people with a family history of MDD.

Because the reports of childhood abuse were retrospective and subjective, we conservatively designated scores in the upper tertile of the distribution as reflecting a higher likelihood of abuse. By way of providing criterion validity for this procedure, Table 2 presents published cut scores that were developed to identify individuals who were most likely to have been physically abused or neglected in childhood [26, 27]. The cut scores capture the most extreme 5–10% of a normative distribution of scores and have a specificity and sensitivity of 98% and <50%, respectively, based on a trauma history interview as the index of criterion validity. Also appearing in Table 2 are the average values for people in the current sample that scored in the upper tertile group. The average values for people scoring in the upper tertile for the physical and emotional abuse and physical and emotional neglect scales felt at or near the normative cut points, suggesting a high likelihood of severe to extreme abuse in this group.

### 3.1. Joint Effects of MDD Family History and Childhood Abuse

**Figure 1:**
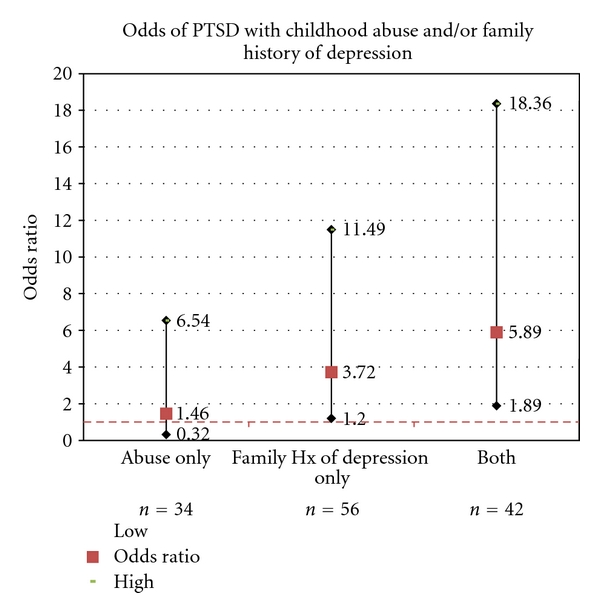
Odds ratios for risk of developing PTSD in the context of a family history of major depressive disorder and childhood abuse.

The rate of PTSD in the reference group was 5.6%, followed by 8.8% in the group with childhood trauma only, 19.6% in the group with a family history of MDD only and 28.6% in people exposed to both factors. Results from the logistic regression analyses showed that compared to the reference group, a history of childhood abuse by itself was not associated with a diagnosis of PTSD in the absence of a family history of MDD (OR = 1.46, *P* = .62), but MDD family history alone resulted in a nearly four-fold risk relative to the comparison group (OR = 3.72, *P* = .02). Notably, the rate of PTSD in the presence of both factors was almost six-fold (OR = 5.89, *P* = .001) relative to the comparison group (see Figure 1). The most common forms of trauma exposure included rape (*n* = 8); chronic childhood abuse of any kind (*n* = 7); beaten/attacked by stranger (*n* = 4); chronic adult physical abuse by a domestic partner (*n* = 3); observed or heard about sudden or violent death of a close friend or relative (*n* = 3); military service, including military sexual trauma (*n* = 2). Analyses were repeated excluding the 7 individuals who reported chronic childhood abuse, and the results were not changed appreciably as the rate of PTSD in people who had both exposures was nearly five-fold (OR = 4.67, *P* = .02). Additional analyses were also conducted including a variable denoting the number of Axis II diagnoses to evaluate whether severity could account for the joint association between abuse history and family history of MDD for PTSD. These analyses showed that the number of Axis II diagnoses did not account for the significant association between the joint effect of childhood abuse and family history of MDD on PTSD as the association continued to be statistically significant (OR = 6.22, *P* = .002).

**Figure 2:**
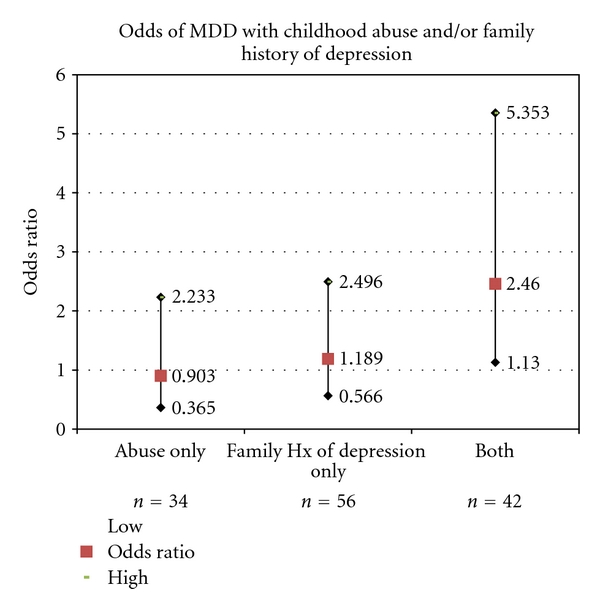
Odds ratios for risk of developing MDD in the context of a family history of Major Depressive Disorder and childhood abuse.

For MDD, the rate in the reference group was 26.7%, followed 26.5% in the group with childhood trauma only, 32.1% in the group with a family history of MDD only, and 50% in people exposed to both factors. The rate of MDD in the presence of childhood abuse or family history of MDD alone was not significantly greater than the reference group (OR's ~= 1, ns), but the presence of both factors was associated with a nearly three-fold risk relative to the reference group (OR = 2.75, *P* = .01) (see Figure 2).

## 4. Discussion

The results presented here contribute to our understanding of risk for PTSD and show that the joint effect of familial history of MDD and childhood trauma exposure significantly increases the rate of PTSD and, although less strongly, of MDD, in people with DSM-IV Axis II disorders. Childhood abuse by itself was not associated with elevated rates for either disorder and appears to require the co-occurrence of familial MDD, at least in the context of this sample of people with Axis II psychopathology. Although this result might seem unexpected based on the past literature (e.g., [28]), the absence of such an association is likely due to the high degree of overlap between depression family history and abuse in this sample as only 15% of the total sample reported childhood trauma exposure in the absence of familial depression. Therefore, we likely did not have adequate power to detect this association. Additionally, the recruitment strategy likely resulted in a biased sample with respect to MDD, as people with current diagnoses were excluded from participation in the research program.

The results also contribute to a large body of research documenting the adverse effects of a family history of depression, including age of onset, severity, and prognosis of MDD [11, 29], an elevated rate of comorbid psychiatric conditions [30, 31], poorer social adjustment, and higher rate of self-reported physical illnesses [31]. Results reported here showed that familial depression by itself was associated with higher rates of PTSD, consistent with observations that people with PTSD report a family history of depressive illness [13, 14]. Depression history was also associated with childhood physical neglect (e.g., “I did not have enough to eat”), childhood sexual abuse, and childhood emotional abuse (e.g., “I thought that my parents wished I had never been born”), lending support to the view that parenting behaviors may influence the development of psychopathology. For example, Johnson and colleagues [19] reported that maladaptive parenting behaviors mediated the association between parental psychopathology and the development of psychopathology in offspring in a longitudinal design.

The current study design does not allow for causal inferences and the manner of interfamilial transmission of PTSD is understudied. Parenting practices are related to parental temperament, suggesting heritable influences on behavior. Moreover, parental behavior is reciprocally influenced by offspring temperament and behavior [32]. Another contributor to the development of PTSD is parental PTSD symptoms, which can influence parental behaviors toward children generally (e.g., disturbed attachment) and in response to trauma exposure (e.g., avoidance of discussing or processing trauma cues) [33]. For example, Lieberman et al. [34] has reported that maternal PTSD mediated the relationship between the level of maternal life stress and child behavior problems. Familial diagnoses of PTSD were not made in the current study, precluding the evaluation of this family history variable on proband diagnoses of PTSD, but such mechanisms should be examined in future work.

There are a number of limitations of the current analyses. First, the family history interview method that was used here was based on a single report (the proband), which was not corroborated with additional reports. Family history data can be biased according to the proband's diagnosis, gender, and age [11]. All participants in the current study were diagnosed with Axis II personality disorders and in order to minimize the influence of proband's diagnosis on reports of symptoms, the interviewers were unaware of the proband's diagnosis at the time of the interview. Additionally, age was unrelated to family history of depression, although as might be expected given the association between gender and the prevalence of MDD, a family history of depression was more common among women in the sample. The fact that the study cannot be generalized to the larger population of people with personality disorders is also a limitation of this study, given that exclusion criteria for participation in the research program included current and significant medical illness, a current diagnosis of major depression, substance abuse or dependence. We opted to report the joint effects of family history of MDD and childhood abuse on proband diagnoses of MDD despite the exclusion of people with a current MDD diagnosis for comparison purposes.

It should also be noted that no associations between specific types of childhood abuse (e.g., physical and sexual neglect), and adult psychiatric disorders were tested as the measure of childhood abuse used in the current study was a total score on a self-report measure of recalled abuse. The total score was chosen for analyses because of the frequent co-occurrence of different types of abuse in the same person [35–37]. Moreover, given the lack of a clear association between a specific type of abuse (e.g., physical versus sexual abuse) and PTSD in the literature, a specific prediction about specificity between abuse type and PTSD is not warranted at this time. With respect to the limitations of retrospective reporting, validity studies suggest that although specific details about abuse episodes may be lost or distorted, people generally can recall whether any abuse occurred or not and false positive reports are rare [26, 27, 37]. Nevertheless, we acknowledge that retrospective reports of abuse can be biased, and for this reason, we set conservative criteria for likelihood of abuse and note that the average values observed in this group fell at the level of published norms suggesting the presence of extreme abuse. Also, people with a family history of MDD did not report more socially desirable responses on the CTQ than people not reporting a family history of depression. Finally, the current analyses were conducted in a sample of people with Axis II personality disorders, which affects the generalizability of the findings to the population at large. The rate of PTSD in the full sample was nearly 14% and MDD was 32%, which are higher than population prevalence estimates.

In sum, the joint effect of abuse history and familial depression was an increased rate of PTSD in a sample of people with Axis II disorders. Results further document the intergenerational transmission of psychopathology and suggest that treatment of depression, particularly when combined with treatment approaches that address maladaptive parenting practices, could alleviate distress and impairment in individuals specifically, and in families, more generally.

## Figures and Tables

**Table 1:** Childhood trauma questionnaire subscale values by family history of depression.

	−Family history MDD	+Family history MDD	*t*	*P*
Emotional abuse	11.27 (5.32)	13.35 (5.53)	−2.83	.005
Physical abuse	8.79 (4.36)	9.61 (4.75)	−1.34	.18
Sexual abuse	7.24 (4.47)	9.00 (5.50)	−2.56	.01
Emotional neglect	12.67 (5.08)	13.58 (5.64)	−1.25	.21
Physical neglect	10.27 (2.12)	11.39 (3.04)	−3.10	.002
Total childhood abuse	55.1 (14.70)	61.06 (17.30	−2.28	.006
Minimization/denial	0.23 (0.68)	0.18 (0.50)	1.39	.61

**Table 2:** Childhood trauma questionnaire normative cut scores for severe-to-extreme abuse.

	Cut score	Upper tertile
Emotional abuse	≥16	17.41 (4.53)
Physical abuse	≥13	13.21 (5.03)
Sexual abuse	≥13	11.47 (6.48)
Emotional neglect	≥18	17.57 (4.38)
Physical neglect	≥13	12.39 (3.25)
